# Efficacy of Electromyographic Biofeedback in the Recovery of the Vastus Lateralis after Knee Injury: A Single-Group Case Study

**DOI:** 10.3390/muscles2040028

**Published:** 2023-11-07

**Authors:** Verónica Morales-Sánchez, Rafael E. Reigal, Raul Antunes, Rui Matos, Antonio Hernández-Mendo, Diogo Monteiro

**Affiliations:** 1 Social Psychology, Social Work and Social Services and Social Anthropology, University of Málaga, 29071 Málaga, Spain; vomorales@uma.es (V.M.-S.); rafareigal@uma.es (R.E.R.); mendo@uma.es (A.H.-M.); 2 ESECS-Polytechnic of Leiria, 2411-901 Leiria, Portugal; raul.antunes@ipleiria.pt (R.A.); rui.matos@ipleiria.pt (R.M.); 3 Life Quality Research Centre (CIEQV), 2040-413 Rio Maior, Portugal; 4 Center for Innovative Care and Health Technology (ciTechCare), Polytechnic of Leiria, 2411-901 Leiria, Portugal; 5 Research Center in Sport Sciences, Health Sciences and Human Development (CIDESD), 5001-801 Vila Real, Portugal

**Keywords:** electromyographic biofeedback, retraining, muscle rehabilitation, knee, patellar tendon, meniscus

## Abstract

Electromyographic biofeedback (EMG-BF) is a technique that can contribute to the improvement of muscle tone and control in the rehabilitation process after injury. The aim of this research was to determine the effectiveness of EMG-BF in increasing the electromyographic activity of the vastus lateralis after knee injury. The sample consisted of four individuals who had undergone surgery or rehabilitation to resolve either a partial meniscal tear or a patellar tendon strain. The intervention consisted of a program of ten sessions of EMG-BF work. Twelve trials were performed in each session, in which participants were instructed to target the muscle tension produced by the vastus lateralis of the uninjured hemilateral limb. Of the twelve trials in each session, the first three and the last three were performed without feedback, and the intermediate six with feedback. The recording of muscle activity was performed using CY-351/2 Mioback equipment, which allowed the amplitude of the electromyographic signal to be evaluated. The results indicated that the sample analyzed reached greater amplitude during the biofeedback trials, both for the maximum (Z = −13.43, *p* < 0.001, Cohen’s d = 0.64, 95% CI (0.27, 1.01)) and mean (Z = −7.26, *p* < 0.001, Cohen’s d = 0.24, 95% CI (−0.12, 0.60)) values. The amplitude also increased throughout the ten sessions, both for the maximum (Z = −3.06, *p* < 0.01, Cohen’s d = 1.37, 95% CI (0.29, 2.45)) and mean (Z = −3.06, *p* < 0.01, Cohen’s d = 1.20, 95% CI (0.34, 2.08)) values. Thus, the results highlight the efficacy of this technique in improving muscle activity, suggesting that it is a useful therapeutic procedure in injury recovery.

## 1. Introduction

Biofeedback (BF) is a technique that monitors autonomic and physiological responses, allowing the control and improvement of this response [1,2]. BF is an audiovisual feedback system linked to the autonomic or physiological signal produced when a person begins to exercise voluntary control over that response [3]. For this purpose, these devices incorporate displays that are configured to give information of a graphical and/or numerical nature that helps to establish voluntary control over the response [4]. Specifically, electromyographic biofeedback (EMG-BF) is a technique that allows for electrical information to be collected from the muscle [5,6]. Thus, depending on the degree of excitation of the muscle, the transmitted signal will be more or less intense, which is generally displayed acoustically and/or visually by devices that translate the electrical information received [7]. EMG-BF is a non-invasive procedure based on the use of surface electrodes to collect electrical activity from the muscle [8]. This allows patients to self-monitor the response by learning the sensations associated with a certain level of contraction intensity [9].

EMG-BF is a widely used technique for the rehabilitation of injuries affecting muscular activity, both in sports and in other contexts involving people’s daily lives [10,11,12,13]. For example, its efficacy has been observed in musculoskeletal readaptation after locomotor system injuries, as well as in recovery processes after neurological injuries that affect movement [14,15]. For example, Liang et al. [14] carried out a study on a sample of women. A postpartum program was conducted in which one group received electromyographic biofeedback pelvic floor muscle training in combination with neuromuscular electrical stimulation of the rectus abdominis, and another group received only neuromuscular electrical stimulation. The results showed a decrease in inter-recti distance for the group that received both interventions. Florjanski et al. [15] showed the ability of EMG-BF to regulate masticatory muscle activity, reducing chronic muscle pain, myofascial pain, myofibrotic contracture, headache, and other pains. 

Thus, when there is muscle involvement, this type of procedure is very useful for regulating muscle activity and recovering movement control [16]. This technique has been shown to be effective after bone, ligament, or muscle injuries that directly or indirectly affect muscle function [17,18,19,20,21]. For example, Hernández-Mendo [17] evaluated the efficacy of EMG-BF in increasing the electromyographic activity of the vastus lateralis in professional soccer players after knee injury, showing the positive effects of a two-week program with EMG-BF in which biofeedback sessions alternated with rest days. Likewise, Christanell et al. [21] analyzed a sample of sixteen people who had undergone endoscopic anterior cruciate ligament reconstruction. They showed that a program which included EMG-BF was better than only a standard program in improving the function of vastus medialis and knee extension.

When practicing physical activity and sports, the prevalence of lower limb injuries is high [22,23]. Due to the permanent stress that these limbs endure, it is common for muscular or osteoarticular injuries to occur in the legs, hips, knees, or ankles [23,24]. When this occurs, there is a period of recovery and readaptation for the affected limb to re-establish itself [25,26,27,28]. Specifically, the knee is associated with a wide variety of injuries, both in sports and in other, more everyday contexts [29,30]. After a traumatic event, the knee can present two important injuries, which include patellar tendon strain and partial meniscal tear, among others [31,32]. A patellar tendon strain is a common injury that requires physiotherapeutic treatment and complementary muscle strengthening measures after a previous period of inactivity to reduce pain and reduce the structural damage generated in the tendon [31,33]. A partial meniscal tear can be more or less serious depending on the severity of the damage and whether it occurs in the medial or lateral area [34]. Surgical intervention to repair this type of injury, such as meniscectomy, is very common in both the general and sports population [35,36,37,38,39,40].

These types of injuries cause the temporary cessation of locomotor activity and musculature associated with knee movement and may cause a loss of functional capacity, which requires a work-up process to improve performance [41,42,43,44]. For this, different procedures are available, among which is EMG-BF. Previous research has observed that this technique is suitable for recovering the control and functional capacity of affected musculature after injury [18,19,20]. For example, Hernández-Mendo and Morales-Sánchez [18] showed the efficacy of EMG-BF when used on professional soccer players with various injuries, such as radius fracture or partial meniscus tear to increase muscle activity in various muscles such as the brachioradialis or the vastus lateralis. In addition, Draper and Ballard [19] conducted an investigation on thirty people who had undergone ACL reconstruction. They observed that the six-week program combining EMG-BF and isometric exercise was more effective in regaining strength in the quadriceps femoris musculature than a program without EMG-BF. These authors indicated that the group rehabilitated with biofeedback recovered a higher percentage of maximum contractile capacity. Likewise, Karaborklu-Argut et al. [20] carried out a systematic review in which they analyzed studies that evaluated the effectiveness of EMG-BF in aiding rehabilitation after knee surgeries. This review highlighted that most research concluded that EMG-BF was more effective than other standard rehabilitation programs in improving the strength and functionality, as well as reducing the pain, of the quadriceps femoris musculature.

When intending to use a therapeutic technique, it is essential to validate its efficacy. Although biofeedback has previously been used to improve the recovery of various injuries, it is difficult to conduct research with a specific clinical population due to its scarcity. The use of EMG-BF following an injury and on a specific muscle is a subject of study with limited prevalence. In the present study, we specifically analyzed two types of injuries that have a significant impact on the physical inactivity of those affected, which generates an important focus of interest when analyzing the efficacy of this technique. Thus, the purpose of this study was to analyze the efficacy of EMG-BF in improving electromyographic activity in the vastus lateralis in a sample that had undergone surgery after a partial meniscus fracture or patellar tendon strain. For this purpose, a multiple-case study is presented that reproduces the efficacy criteria proposed by Chambless and Hollon [45]. Likewise, the choice of the vastus lateralis is justified because it is one of the muscles that provides the most stability to the knee, as pointed out by authors such as Jarvela et al. [46].

## 2. Materials and Methods

### 2.1. Design

This research uses an intrasubject quasi-experimental design (A → B → A), with nonrandom assignment and pretest (baseline) and posttest measures. The intervention protocol is registered at https://www.clinicaltrials.gov/ (accessed on 29 April 2022), with identifier NCT05376072. In addition, the protocol used in this research conforms to the so-called BFB (biofeedback) training [47], in which muscle activity is voluntarily modified after obtaining information about previous contractions.

### 2.2. Participants

Four people participated in this research. 

Case 1: This is a 20-year-old basketball player who plays for an amateur team and competes at a local level. Due to a patellar tendon strain, he was inactive for a period of 10 months. The etiology of the injury itself caused a loss of muscle tone in the quadriceps of the left leg. To alleviate this loss of tone, the medical prescription consisted of concentric and eccentric strength exercises at least three times a week. 

Case 2: This is a 21-year-old soccer player diagnosed with a partial meniscus tear in his right leg. As a consequence, he underwent a partial meniscectomy thirty days prior to the investigation. Before undergoing biofeedback treatment, he received laser therapy and practiced functional recovery exercises. By medical indication, he attended daily physical rehabilitation sessions. At the time of the biofeedback intervention, he presented slight pain in the operated leg, which intensified with flexion extension exercises of the knee against resistance, as well as hypotrophy of the right quadriceps.

Case 3: This is a 30-year-old woman who is regularly physically active and was diagnosed with a partial meniscus tear in her left leg. As a consequence, she underwent a partial meniscectomy fifteen days prior to the investigation. Post-operatively, she was immobilized for three days and subsequently had to use crutches for a week. Before undergoing biofeedback treatment, she received laser therapy and practiced functional recovery exercises. By medical indication, she attended daily physical rehabilitation sessions.

Case 4: This is a 36-year-old male who is regularly physically active and presented with a diagnosis of partial meniscal tear in the right leg. He underwent partial meniscectomy twenty days prior to the investigation. Post-operatively, he was immobilized for four days and subsequently had to use crutches for a week. By medical indication, he attended daily physical rehabilitation sessions.

### 2.3. Measurements and Instruments

The recording of EMG activity was performed using 15 mm diameter surface and self-adhesive electrodes. A two-channel electromyograph (Mioback CY-351/2, Biociber S.L., Barcelona, Spain) [48] was used to evaluate the different parameters and provide visual and auditory feedback of EMG activity. The electromyograph performs automatic amplification and filtering processes. It has a reading speed of 30 data/second between 0.1 and 20,000 microvolts, and a frequency of between 30 and 300 Hz. Likewise, two splints were used that allowed the maintenance of the knee posture at full extension. In the baseline sessions, the electromyographic activity of the vastus lateralis was evaluated during maximal effort isometric contraction, and measurements were taken, in all cases, of the signal amplitude. In addition, for the correct placement of the electrodes and good reception of the electromyographic signal, the area was shaved and cleaned with ethyl alcohol. The training sessions consisted of twelve trials each, with auditory and visual feedback of vastus lateralis EMG activity collected in all cases. 

### 2.4. Procedure

All participants signed an informed consent form before participating in the research. In addition, the principles of the Declaration of Helsinki [49] were respected throughout the process. Likewise, the study was approved by the ethics committee of the University of Malaga. The inclusion criteria included having suffered a knee injury, either a patellar tendon strain or a partial meniscal tear, which had been resolved by surgery or physiotherapeutic treatment.

The intervention lasted twenty days, during which 10 sessions of EMG-BF work were performed with 1 rest session between each of them. Electromyographic activity was evaluated during an isometric contraction of maximum effort in the vastus lateralis, recording the amplitude of the electromyographic signal in millivolts or microvolts (depending on the saturation of the channel). Each contraction lasted six seconds, and a rest of at least two minutes was allowed between contractions. Each session lasted approximately 20 to 30 min. Also, the work target during the contraction of the injured limb was the maximum intensity previously reached by the uninjured limb, which served as a work criterion for the performer.

Each session was divided into three phases and a total of twelve trials: (a) three trials without feedback, (b) six trials with biofeedback, and (c) three trials without feedback. This session structure allowed intrasession gain (differences between phase a and c) and intercession gain (differences between phase c of the previous session and phase c of the current session) to be assessed. On the other hand, so that the electrodes would always have the same location, a visible mark was made with an indelible marker. For this investigation, the mean and maximum values reached during contractions (amplitude in millivolts/microvolts) were used. 

In all trials, we worked with isometric contractions. The participants remained in a sitting position, with their legs resting on a chair and both knees extended. During isometric contraction, the biofeedback equipment records the amplitude, the mean and maximum of the electromyographic signal, and the contraction and stiffening times. Contraction time is considered to be the time elapsed between the onset of the contraction and the moment when the desired muscle activity is achieved. On the other hand, hardening time is considered to be the time interval after which the tension reached is maintained [50]. 

During exercise with EMG-BF, the system displayed numerical visual and auditory signals through a display which depended on the intensity of the contraction. The auditory information intensified along with the electromyographic signal. According to the signals, and only during the feedback trials, the therapist encouraged the patient to perform the exercise.

### 2.5. Data Analysis 

Descriptive and inferential analyses were performed. Mean, standard deviation, skewness, and kurtosis were calculated. The values of skewness and kurtosis should range from +2 to −2 and +7 to −7, respectively [51]. In addition, the Kolmogorov–Srminov and Shapiro–Wilk tests were used to test for normality, and indicated when the results were not statistically significant. In addition, the Wilcoxon (if normality was not accepted) and Student’s t (if normality was accepted) tests were used to analyze mean differences. SPSS Statistics v.24 software (IBM Corp., Armonk, NY, USA) was used for this purpose. Likewise, the Cohen’s d statistic was used to calculate the effect size (≈0.20: small, ≈0.50: medium, and ≈0.80: large [52]). Analyses of the variance components and generalizability were performed using SAS v.9.1 (SAS Institute Inc., Cary, NC, USA) [53,54] and SAGT v.1.0, respectively (University of Malaga, Malaga, Spain) [55]. An analysis of the variance components was performed using the least squares strategy (VARCOMP Type 1), which is based on decomposing the total variance into related components, and the maximum likelihood strategy (GLM), which is based on seeking values in the model factors that make the observed data more probable [56,57]. Generalizability analysis is a procedure that, after scrutinizing the sources of variation affecting a measurement, provides an estimate of how well the observed mean aligns with the mean of all possible observations. In this analysis, the relative G coefficient is calculated as a measure of reliability, and the absolute G coefficient as a measure of generalizability [58].

## 3. Results

### 3.1. Variance Component Analysis

Initially, a four-facet model was used, but due to the saturation produced by working with a large number of facets, the model without interactions was used initially. The following models were used: (1) [y = p s te ne] for estimating the maximum signal, and (2) the model [z = p s te ne] for estimating the mean signal where p (participant) × s (session) × tt (type of trial) × tn (trial number) − y (maximum EMG signal) z (mean EMG signal). 

In both models, the least squares (VARCOMP Type1) and the maximum likelihood (GLM) strategy were used to check whether the error variances obtained using each procedure were equal ensure that the sample was linear, normal, and homoscedastic [56,57]. It was possible to verify that the error variances obtained using each model—the first model [y = p s tt tn] and the second [z = p s tt tn]—were equal. Thus, for the model [y = p s tt tn], the error variance was VARCOMP Type1= 860 and GLM = 860; for the model [z = p s tt tn], it was VARCOMP Type1 = 978,949 and GLM = 978,948.53. It is assumed, therefore, that the sample used for both models has a linear, normal, and homoscedastic distribution.

It was also found that the model [y = p s te ne] is significant and explains 23.89% of the variance. In addition, all the facets, except te (type of trial), are significant. Similarly, the model [z = p s tt tn] is significant and explains 88.3% of the variance. In addition, all facets, with the exception of n (number of trials), are significant.

Subsequently, four facet models with interaction were estimated: (1) the [y = p × s × tt × tn] model of maximum signal estimation, and (2) the [z = p × s × tt × tn] model of mean signal estimation where p (participant) × s (session) × tt (trial type) × tn (trial number) − y (maximum EMG signal) z (mean EMG signal).

It could be verified, as in the previous case, that the error variances obtained using each model ([y = p × s × tt × tn] and [z = p × s × tt × tn]) were the same. Thus, for the first model, the error variance was VARCOMP Type1 = 860 and GLM = 860; for the second model, it was VARCOMP Type1 = 978,949 and GLM = 978,948.53. It is assumed, therefore, that the sample used for both models has a linear, normal, and homoscedastic distribution. The model [y = p × s × tt × tn] is not significant and collapses all facets because the [tt] facet explains all the variance associated with it. The model [z = p × s × tt × tn] is significant and explains 92.30% of the variance. Similarly, all the facets are significant. All interactions are also significant except for the [p × tn] and [p × s × tn] interactions. And the interactions [tt × tn], [p × tt × tn], [s × tt × tn], and [p × s × tt × tn] collapse due to the contribution of p (participant) and tt (trial type).

### 3.2. Generalizability Analysis

Using the analysis of the variance components of the means [z = p × s × tt × tn] and the sum of squares, a generalizability analysis was performed for this model. A four-facet cross-facet analysis was performed where each of the facets was used sequentially as an instrumentation facet.

A previous analysis of variance was performed where it can be observed (Table 1) that the highest percentage of variance is associated with the facets [p] (participant), which accounts for 69.532%, and [tt] (type of trial).

The results of the generalizability analysis (Table 2) show that for all the models, the relative and absolute G indices (indicating the reliability of the model and its generalizability, respectively) are optimal. The lowest values (0.555 and 0.866) correspond to the model [s] [tt] [tn]/[p] and to the model [p] [s] [tn]/[tt], respectively, where [p] and [tt] act as instrumentation facets (the facet to be estimated) and the facets [s] [tt] [tn] and [p] [s] [tn] act as differentiation facets to the instrumentation facets, respectively. These values can be explained by considering that facets [p] and [tt] have the highest percentages of associated variance.

### 3.3. Trials with BF vs. Trials without BF and Trials before BF vs. Trials after BF

Table 3 shows the descriptive statistics for the mean and maximum electromyographic activity (signal amplitude expressed in microvolts -µV-) for the pre-BF and post-BF tests and for trials without biofeedback, performed during the 10 sessions as a whole. The Shapiro–Wilk test values are also expressed.

**Figure 1:**
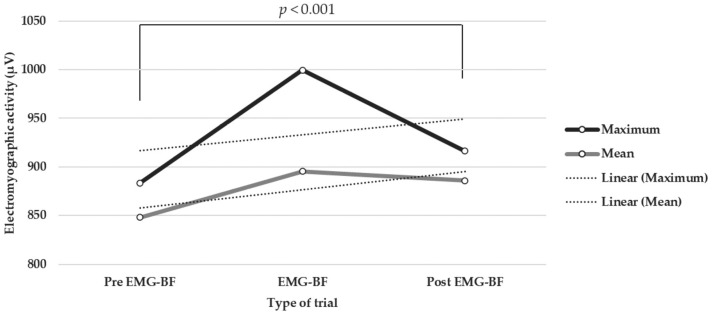
Electromyographic signal differences during the work sessions.

The Wilcoxon tests that were performed indicated the existence of statistically significant differences between trials with EMG-BF and trials without EMG-BF in both their peak (Z = −13.43, *p* < 0.001, Cohen’s d = 0.64, 95% CI (0.27, 1.01)) and mean (Z = −7.26, *p* < 0.001, Cohen’s d = 0.24, 95% CI (−0.12, 0.60)) values. As can be observed, the Cohen’s d values show that the difference between the peak values was greater than that between the mean values. In addition, statistically significant differences were found between pre- and post-test values with EMG-BF in both their peak (Z = −3.83, *p* < 0.001, Cohen’s d = 0.25, 95% CI (−0.25, 0.76)) and mean (Z = −4.48, *p* < 0.001, Cohen’s d = 0.29, 95% CI (−0.22, 0.80)) values (Figure 1). As can be observed, Cohen’s d values show low effects.

### 3.4. Session One vs. Session Ten

Table 4 shows the descriptive statistics (mean, standard deviation, skewness, and kurtosis) of the maximum and mean electromyographic activity values for the trials with and without EMG-BF, as well as the mean of all trials in sessions one and ten. Electromyographic activity is expressed in microvolts (µV).

Wilcoxon tests indicated that there were statistically significant differences between the electromyographic activity recorded in sessions one and ten in in trials without biofeedback, both for the peak (Z = −2.20, *p* < 0.05, Cohen’s d = 1.68, 95% CI (0.36, 2.99)) and mean (Z = −2.20, *p* < 0.05, Cohen’s d = 1.76, 95% CI (0.43, 3.09)) values, as well as in all trials for the maximum (Z = −3.06, *p* < 0.01, Cohen’s d = 1.37, 95% CI (0.29, 2.45)) and mean (Z = −3.06, *p* < 0.01, Cohen’s d = 1.20, 95% CI (0.34, 2.08)) values. Likewise, the Student’s *t* tests performed indicated statistically significant differences between sessions one and ten for the maximum (t = −11.09, *p* < 0.001, Cohen’s d = 4.91, 95% CI (2.65, 7.19)) and mean (t = −2.34, *p* < 0.05, Cohen’s d = 1.55, 95% CI (0.26, 2.84)) values of the trials with biofeedback. As can be observed, the Cohen’s d values show high effects in all cases.

**Figure 2:**
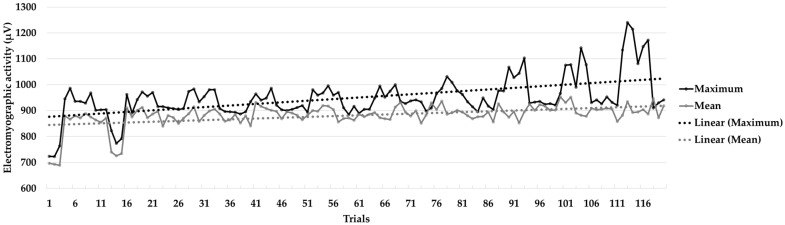
Learning curve throughout the intervention program (120 trials). The graph shows the mean and maximum amplitude values of the electromyographic signal, as well as the trend lines.

Figure 2 contains a graph that shows the learning curve with the trend line. As can be seen, the figure represents a progressive increase in the amplitude of the electromyographic signal collected, as shown by the statistical tests performed.

## 4. Discussion

The purpose of this research was to analyze the efficacy of an EMG-BF intervention in the recovery of quadricep contractile capacity, specifically on the vastus lateralis muscle, in a sample of patients who had suffered a knee injury. Specifically, the study participants had undergone surgical or physiotherapeutic treatment to resolve one of two possible injuries: a partial meniscal tear or a patellar tendon strain. The results show increases in the electromyographic activity of the vastus lateralis during the trials with EMG-BF and throughout the intervention program.

First, the results show statistically significant differences between the trials with and without biofeedback, especially regarding the maximum peaks, for which the effect size was greater. In addition, a significant intra-session effect caused by the EMG-BF intervention was observed, although this showed a low effect size. This occurred because the intrasession comparison is biased by the learning accumulated throughout the sessions. In fact, as seen in the results, the differences between the first and last sessions are highly significant with a large effect size. This suggests that biofeedback increases the ability to regulate muscle activity, as well as increasing contractile capacity and performance. These results are consistent with previous literature, in which various authors have pointed out that the EMG-BF is a useful procedure for improving the readaptation of the muscles involved in a process of osteoarticular injury [10,11,12,13,14,15,17,18,19,20,21,22]. As an example, this intra-session effect has been previously reported in research carried out by Morales-Sánchez et al. [59], in which gains in electromyographic activity were observed after an intervention with EMG-BF in a sample undergoing surgery to resolve a partial meniscus tear.

Second, this effect was observed throughout the program. The results show a positive learning curve in both the average and maximum electromyographic activity throughout the entire process. In addition, statistically significant differences can be seen between the first and the last session of the program, and a large effect size was observed. This highlights the effectiveness of the training and suggests that electromyographic biofeedback is a suitable technique for improving muscle control and strength, as previous studies have shown [4,10,11,22,47]. Similar results have been observed in previous studies on samples with knee injuries, with gains in muscle activity being observed as a result of programs in which EMG-BF was implemented [17,18,19,20,21]. Specifically, our findings coincide with the effects found by Hernández-Mendo and Morales-Sánchez et al. [17,18,55], who used similar intervention programs. It is more difficult to compare these findings with those obtained by other investigators using control group designs [19,20,21]. In this research, an intragroup design was used. However, the evidence obtained in these investigations is consistent with that presented here, since an increase in the electromyographic activity caused by the EMG-BF was observed and its usefulness in this type of lesions can be inferred.

These results reflect the efficacy of a technique that can be used during injury recovery processes in physical activity and sports practitioners. Therefore, EMG-BF is proposed as useful for improving these processes and increasing the likelihood of recovery, improving muscle readaptation. Furthermore, in injuries such as patellar tendon strain or partial meniscal tear, it is important that the musculature adjacent to the knee recover well, given that they serve as supporting musculature for the articular structure [31,32]. Therefore, even if other physiotherapeutic techniques are used for this type of injury, it is suggested that EMG-BF may contribute to a successful recovery [18,19,20]. 

### 4.1. Practical Applications

These results have important practical applications. As is known, during these periods of inactivity, there is a decrease in muscle functionality, which results in difficulties regarding physical readaptation and returning to physical sports practice [33,41,42,43,44]. Injured people lose motor sensitivity and contractile capacity in the muscles involved. Therefore, it is considered appropriate to use techniques that improve the effectiveness of rehabilitation. For this reason, and based on the scientific results, it is suggested that EMG-BF be used in the early phases of the recovery process in addition to other standard osteoarticular and muscular rehabilitation procedures. Therefore, implementing intervention programs with EMG-BF in the first weeks after the injury would improve the functionality of the muscle and speed up its recovery.

### 4.2. Limitations

The present study has a number of limitations. First, there was no follow-up of the gains obtained by EMG-BF, which makes it impossible to determine to what extent the learning obtained through biofeedback is maintained in the future. It would be interesting to carry out follow-up sessions with biofeedback to check if, after an intervention with ten sessions of EMG-BF, this learning is consolidated. In the present study, all the sessions were carried out with biofeedback, so we do not know whether subsequent sessions carried out without this procedure allow the injured person to maintain the contractile capacity of the muscle that has been worked on. Secondly, this research has been performed on the vastus lateralis muscle, but we do not know what effect it could have had on another muscle section. In addition, it would also be interesting to determine whether there is a transfer of the learning generated to other muscles. This would be very interesting, since it would increase the performance of this technique and its therapeutic capacity. Thirdly, it would be interesting to test the efficacy of this technique alongside other therapeutic interventions. However, the difficulty involved in finding clinical muscles with this type of lesion complicates this possibility. Fourth, more studies should be carried out in the future with a design that includes a control group and a larger number of participants. Furthermore, future research could be carried out by modifying the range of motion during contraction to analyze how this variable affects the evolution of the contractile capacity of the muscle.

In any case, the results shown in this study highlight the efficacy of EMG-BF in the improvement of vastus lateralis muscle control after a patellar tendon strain or a partial meniscal tear, suggesting that it may be a useful therapeutic tool in these cases.

## 5. Conclusions

These findings suggest that BF-EMG is an interesting component to be considered for use in conjunction with other therapeutic procedures, since it can contribute to better muscle readaptation and the more accurate recovery of pre-injury muscle tone. Given that this type of injury generates a significant period of inactivity, the acceleration of the contractile capacity of the muscle is an issue to be taken into account. For this reason, BF-EMG could be used in meniscus and patellar tendon injuries to improve the muscular control of the quadriceps musculature. Based on the results presented here, twenty-day programs with ten work sessions which include BF-EMG would be appropriate for a substantial improvement in the recovery of the muscle tone of the vastus lateralis, which is useful for improving the performance of this muscle.

## Figures and Tables

**Table 1:** Analysis of variance of the model [z = p × s × tt × tn].

Sources of Variation	Sum of Squares	Degree of Freedom	Mean Square	%
[p]	8,394,421.45	3	2,798,140.48	69.53
[s]	955,988.74	9	106,220.97	3.82
[p][s]	210,894.61	27	7810.91	0
[tt]	1,191,352.64	1	1,191,352.64	14.15
[p][tt]	117,122.04	3	39,040.68	1.53
[s][tt]	330,312.53	9	36,701.39	3.53
[p][s][tt]	233,506.63	27	8648.39	4.35
[tn]	64,863.69	2	32,431.85	0.43
[p][tn]	1048.08	6	174.68	0
[s][tn]	192,699.31	18	10,705.52	1.82
[p][s][tn]	58,809.88	54	1089.07	0.82
[tt][tn]	0	2	0	0
[p][tt][tn]	0	6	0	0
[s][tt][tn]	0	18	0	0
[p][s][tt][tn]	0	54	0	0

Note. p = participant; s = session; tt = type of trial; tn = trial number).

**Table 2:** Results of the generalizability analysis of the model [z = p|s|tt|tn].

Face	Levels	Size Universe	Description	% Variance	Model Generalizability	G Relative	G Absolute
[p]	4	INF	participants	69.53	[s] [tt] [tn]/[p]	0.934	0.555
[s]	10	INF	session	3.82	[p] [tt] [tn]/[s]	0.988	0.984
[tt]	2	INF	type of test	14.15	[p] [s] [tn]/[tt]	0.942	0.866
[tn]	3	INF	test number	0.42	[p] [s] [tt]/[tn]	0.991	0.990

Note. p = participant; s = session; tt = type of trial; tn = trial number).

**Table 3:** Descriptive statistics for maximum and mean electromyographic activity values for the trials with and without biofeedback, as well as before and after BF.

	Electromyographic Activity (µV)					
	Values	M	SD	S	K	S-W
Pre-EMG-BF trials	Maximum	883.54	135.57	−1.03	0.03	0.29 ***
	Mean	848.34	135.22	−0.92	−0.18	0.25 ***
Post-EMG-BF trials	Maximum	916.41	123.06	−1.23	−0.25	0.14 ***
	Mean	885.83	120.01	−1.21	−0.01	0.26 ***
EMG-BF trials	Maximum	999.61	176.97	−0.13	−0.10	0.36 ***
	Mean	895.39	110.10	−1.18	0.174	0.30 ***
Without EMG-BF trials (pre and post)	Maximum	899.98	130.23	−1.12	−0.04	0.33 ***
	Mean	867.08	128.95	−1.06	−0.07	0.28 ***

Note. M = mNote. M = Mean; SD = standard deviation; S = skewness; K = kurtosis; µV = microvolts; S-W= Shapiro–Wilk. *** p < 0.001.

**Table 4:** Descriptive statistics for maximum and mean electromyographic activity values for trials with and without biofeedback in sessions one and ten, as well as the mean of all trials.

		Electromyographic Activity (µV)					
	Session	Values	M	SD	S	K	S-W
Without EMG-BF trials	1	Maximum	819.90	91.94	−0.10	−3.08	0.76 *
		Mean	778.34	93.38	00.01	−3.29	0.73 *
	10	Maximum	930.82	15.28	0.11	−0.29	0.99
		Mean	901.60	32.04	−0.12	−0.64	0.96
EMG-BF trials	1	Maximum	950.08	22.58	1.02	−0.48	0.86
		Mean	876.66	7.71	0.26	0.17	0.99
	10	Maximum	1165.05	57.54	−0.10	−0.59	0.98
		Mean	898.91	18.79	1.68	3.10	0.84
All trials (mean)	1	Maximum	884.99	93.25	−1.07	−0.37	0.80 *
		Mean	827.50	81.40	−1.29	−0.36	0.65 ***
	10	Maximum	1047.94	128.74	0.27	−1.87	0.83 *
		Mean	900.26	25.08	0.26	−0.09	0.98

Note. M = mean Note. M= Mean; SD = standard deviation; S = skewness; K = kurtosis; µV = microvolts. * p < 0.05; *** p < 0.001.

## Data Availability

Data are available upon request from the authors.

## References

[1] Abril-Rodríguez S., Herrero R. (2022). Biofeedback electromiográfico y electroglotográfico aplicado a la terapia vocal: Una revisión sistemática. Rev. Investig. Logopedia.

[2] Duarte-Moreira R.J., Castro K.V.F., Luz-Santos C., Martins J.V.P., Sá K.N., Baptista A.F. (2018). Electromyographic biofeedback in motor function recovery after peripheral nerve injury: An integrative review of the literature. Appl. Psychophys. Biof..

[3] Giggins O.M., Persson U., Caulfield B. (2013). Biofeedback in rehabilitation. J. Neuroeng. Rehabil..

[4] Lehrer P., Kaur K., Sharma A., Shah K., Huseby R., Bhavsar J., Sgobba P., Zhang Y. (2020). Heart rate variability biofeedback improves emotional and physical health and performance: A systematic review and meta analysis. Appl. Psychophys. Biof..

[5] Chowdhury R.H., Reaz M.B.I., Ali M.A.M. (2013). Surface Electromyography Signal Processing and Classification Techniques. Sensors.

[6] De Luca C.J., Webster J.G. (1988). Electromyography. Encyclopaedia of Medical Devices and Instrumentation.

[7] Phinyomark A., Campbell E., Scheme E., Naik G. (2020). Surface Electromyography (EMG) Signal Processing, Classification, and Practical Considerations. Biomedical Signal Processing: Advances in Theory, Algorithms and Applications.

[8] Jaramillo-Yánez A., Benalcázar M.E., Mena-Maldonado E. (2020). Real-Time Hand Gesture Recognition Using Surface Electromyography and Machine Learning: A Systematic Literature Review. Sensors.

[9] McGill K. (2004). Surface electromyogram signal modelling. Med. Biol. Eng. Comput..

[10] Sklempe-Kokic I., Vuksanic M., Kokic T., Peric I., Duvnjak I. (2022). Effects of Electromyographic Biofeedback on Functional Recovery of Patients Two Months after Total Knee Arthroplasty: A Randomized Controlled Trial. J. Clin. Med..

[11] Jing G. (2018). Clinical effect evaluation and experience of motomed virtual scene training combined with electromyographic biofeedback therapy in the treatment of spastic cerebral palsy. Ann. Phys. Rehab. Med..

[12] Kamonseki D.H., Calixtre L.B., Barreto R.P.G., Camargo P.R. (2021). Effects of electromyographic biofeedback interventions for shoulder pain and function: Systematic review and meta-analysis. Clin. Rehab..

[13] Raeissadat S.A., Rayegani S.M., Sedighipour L., Bossaghzade Z., Abdollahzadeh M.H., Nikray R., Mollayi F. (2018). The efficacy of electromyographic biofeedback on pain, function, and maximal thickness of vastus medialis oblique muscle in patients with knee osteoarthritis: A randomized clinical trial. J. Pain Res..

[14] Liang P., Liang M., Shi S., Liu Y., Xiong R. (2022). Rehabilitation programme including EMG-biofeedback-assisted pelvic floor muscle training for rectus diastasis after childbirth: A randomised controlled trial. Physiotherapy.

[15] Florjanski W., Malysa A., Orzeszek S., Smardz J., Olchowy A., Paradowska-Stolarz A., Wieckiewicz M. (2019). Evaluation of biofeedback usefulness in masticatory muscle activity management—A systematic review. J. Clin. Med..

[16] Neblett R. (2016). Surface Electromyographic (SEMG) Biofeedback for Chronic Low Back Pain. Healthcare.

[17] Hernández-Mendo A. (2011). Biofeedback electromiográfico en la rehabilitación de lesiones de rodilla. Estudio de dos casos en futbolistas profesionales. Cuad. Psicol. Deporte.

[18] Hernández-Mendo A., Morales-Sánchez V. (2014). Efectividad del biofeedback electromiográfico en la rehabilitación de lesiones deportivas. Rev. Psicol. Deporte.

[19] Draper V., Ballard L. (1991). Electrical stimulation versus electromyographic biofeedback in the recovery of quadriceps femoris muscle function following anterior cruciate ligament surgery. Phys. Ther..

[20] Karaborklu-Argut S., Celik D., Yasacı Z. (2022). Effectiveness of therapeutic electromyographic biofeedback after orthopedic knee surgeries: A systematic review. Disabil. Rehabil..

[21] Christanell F., Hoser C., Huber R., Fink C., Luomajoki H. (2012). The influence of electromyographic biofeedback therapy on knee extension following anterior cruciate ligament reconstruction: A randomized controlled trial. Sports Med. Arthrosc. Rehabil. Ther. Technol..

[22] Xie Y.J., Wang S., Gong Q.J., Wang J.X., Sun F.H., Miyamoto A., Ou X., Wang S.Q., Zhang C. (2021). Effects of electromyography biofeedback for patients after knee surgery: A systematic review and meta-analysis. J. Biomech..

[23] López-Valenciano A., Ruiz-Pérez I., Garcia-Gómez A., Vera-Garcia F.J., Croix M.D.S., Myer G.D., Ayala F. (2020). Epidemiology of injuries in professional football: A systematic review and meta-analysis. Brit. J. Sport Med..

[24] Pérez-Gómez J., Adsuar J.C., Alcaraz P.E., Carlos-Vivas J. (2020). Physical exercises for preventing injuries among adult male footballsoccer players: A systematic review. J. Sport Health Sci..

[25] Morgan J.P.M., Hamm M., Schmitz C., Brem M.H. (2021). Return to play after treating acute muscle injuries in elite football players with radial extracorporeal shock wave therapy. J. Orthop. Surg. Res..

[26] Larruskain J., Lekue J.A., Martin-Garetxana I., Barrio I., McCall A., Gil S.M. (2022). Injuries Are Negatively Associated with Player Progression in an Elite Football Academy. Sci. Med. Footb..

[27] Jones A., Jones G., Greig N., Bower P., Brown J., Hind K., Francis P. (2019). Epidemiology of injury in English Professional Football players: A cohort study. Phys. Ther. Sport.

[28] Read P.J., Oliver J.L., De Ste Croix M., Myer G.D., Lloyd R.S. (2016). Neuromuscular risk factors for knee and ankle ligament injuries in male youth soccer players. Sports Med..

[29] Bollen S. (2000). Epidemiology of knee injuries: Diagnosis and triage. Br. J. Sports Med..

[30] Arundale A.J., Silvers-Granelli H.J., Marmon A., Zarzycki R., Dix C., Snyder-Mackler L. (2018). Changes in biomechanical knee injury risk factors across two collegiate soccer seasons using the 11+ prevention program. Scand. J. Med. Sci. Sport.

[31] Nishida Y., Nishino T., Tanaka K., Onishi S., Kanamori A., Yamazaki M. (2021). An Objective Measure of Patellar Tendon Thickness Based on Ultrasonography and MRI in University Athletes. J. Clin. Med..

[32] Adams B.G., Houston M.N., Cameron K.L. (2021). The epidemiology of meniscus injury. Sports Med. Arthrosc..

[33] Warden S.J., Kiss Z.S., Malara F.A., Ooi A.B.T., Cook J.L., Crossley K. (2007). Comparative Accuracy of Magnetic Resonance Imaging and Ultrasonography in Confirming Clinically Diagnosed Patellar Tendinopathy. Am. J. Sports Med..

[34] Steinbacher G., Alentorn-Geli E., Alvarado-Calderón M., Barastegui D., Álvarez-Díaz P., Cugat R. (2019). Meniscal fixation is a successful treatment for hypermobile lateral meniscus in soccer players. Knee Surg. Sports Traumatol. Arthrosc..

[35] Drobnič M., Ercin E., Gamelas J., Papacostas E.T., Slynarski K., Zdanowicz U., Spalding T., Verdonk P. (2019). Treatment options for the symptomatic post-meniscectomy knee. Knee. Surg. Sports Traumatol. Arthrosc..

[36] Alvarez-Diaz P., Alentorn-Geli E., Llobet F., Granados N., Steinbacher G., Cugat R. (2016). Return to play after all-inside meniscal repair in competitive football players: A minimum 5-year follow-up. Knee Surg. Sports Traumatol. Arthrosc..

[37] Vaquero J., Forriol F. (2016). Meniscus tear surgery and meniscus replacement. Muscles Ligaments Tendons J..

[38] Jacob G., Shimomura K., Krych A.J., Nakamura N. (2020). The Meniscus Tear: A Review of Stem Cell Therapies. Cells.

[39] Marcacci M., Marcheggiani-Muccioli G.M., Grassi A., Ricci M., Tsapralis K., Nanni G., Bonanzinga T., Zaffagnini S. (2014). Arthroscopic meniscus allograft transplantation in male professional soccer players: A 36-month follow-up study. Am. J. Sport Med..

[40] Jeong H.J., Lee S.H., Ko C.S. (2012). Meniscectomy. Knee Surg. Rel. Res..

[41] Lee D.H., D’Lima D.D., Lee S.H. (2019). Clinical and radiographic results of partial versus total meniscectomy in patients with symptomatic discoid lateral meniscus: A systematic review and meta-analysis. Orthop. Traumatol. Sur..

[42] Niering M., Muehlbauer T. (2021). Effects of Physical Training on Physical and Psychological Parameters in Individuals with Patella Tendinopathy: A Systematic Review and Meta-Analysis. Sports.

[43] Lavoie-Gagne O.Z., Korrapati A., Retzky J., Bernstein D.N., Diaz C.C., Berlinberg E.J., Forlenza E.M., Fury M.S., Mehta N., O’Donnell E.A. (2022). Return to Play and Player Performance After Meniscal Tear Among Elite-Level European Soccer Players: A Matched Cohort Analysis of Injuries From 2006 to 2016. Orthop. J. Sport Med..

[44] Núñez-Sánchez F.J., Cabrera F.I.M., Abad F.H., Suárez-Arrones L. (2021). Progressive Rehabilitation of a Professional Soccer Player After an Anterior Cruciate Ligament Reconstruction in Phase 1: Clinical Perspective with Video Demonstration. J. Athl. Train..

[45] Chambless D.L., Hollon S.D. (1998). Defining empirically supported therapies. J. Consult. Clin. Psych..

[46] Jarvela T., Kannus P., Latvala K., Jarvinen M. (2002). Simple measurements in assessing muscle performance after an ACL reconstruction. Int. J. Sports Med..

[47] Criado L., de La Fuente A., Heredia M., Montero J., Albaladejo A., Criado J.-M. (2016). Electromyographic biofeedback training for reducing muscle pain and tension on masseter and temporal muscles: A pilot study. J. Clin. Exp. Dent..

[48] Simón M.A., Bueno A.M. (2009). Psychophysiological Profile in Dyssynergic Defecation Patients: An Individual and Situational Response Specificity Analysis. Appl. Psychophysiol. Biofeedback.

[49] World Medical Association (2013). World medical association declaration of Helsinki: Ethical principles for medical research involving human subjects. J. Am. Med. Assoc..

[50] Solé V., Moliner L. (1988). Metodología para el estudio de la fatiga y la contracción muscular. Rehabilitación.

[51] Byrne B. (2016). Structural Equation Modeling with AMOS. Basic Concepts, Applications, and Programming.

[52] Hojat M., Xu G. (2004). A visitor’s guide to effect sizes: Statistical significance versus practical (clinical) importance of research findings. Adv. Health Sci. Educ. Theory Pract..

[53] SAS Institute (1999). User’s Guide.

[54] Schlotzhauer S.D., Littell R. (1997). SAS System for Elementary Statistical Analysis.

[55] Hernández-Mendo A., Blanco-Villaseñor A., Pastrana J.L., Morales-Sánchez V., Ramos-Pérez F.J. (2016). SAGT: New software for generalizability analysis. Rev. Iberoam. Psicol. Ejerc. Deporte.

[56] Hemmerle W., Hartley H. (1973). Computing maximum likelihood estimates for the mixed AOV Model using the w-transformation. Technometrics.

[57] Searle S., Casella G., McCulloch C. (1992). Variance Components.

[58] Blanco-Villaseñor Á., Castellano J., Hernández Mendo A., Sánchez-López C.R., Usabiaga O. (2014). Aplicación de la TG en el deporte para el estudio de la fiabilidad, validez y estimación de la muestra. Rev. Psicol. Deporte.

[59] Morales-Sánchez V., Falcó C., Hernández-Mendo A., Reigal R.E. (2022). Efficacy of Electromyographic Biofeedback in Muscle Recovery after Meniscectomy in Soccer Players. Sensors.

